# Metabolism-linked methylotaxis sensors responsible for plant colonization in *Methylobacterium aquaticum* strain 22A

**DOI:** 10.3389/fmicb.2023.1258452

**Published:** 2023-10-13

**Authors:** Akio Tani, Sachiko Masuda, Yoshiko Fujitani, Toshiki Iga, Yuuki Haruna, Shiho Kikuchi, Wang Shuaile, Haoxin Lv, Shiori Katayama, Hiroya Yurimoto, Yasuyoshi Sakai, Junichi Kato

**Affiliations:** ^1^Institute of Plant Science and Resources, Okayama University, Kurashiki, Japan; ^2^Japan Science and Technology Agency, Advanced Low Carbon Technology Research and Development Program (JST ALCA), Kawaguchi, Japan; ^3^Graduate School of Agriculture, Kyoto University, Kyoto, Japan; ^4^Graduate School of Integrated Sciences for Life, Hiroshima University, Higashihiroshima, Japan

**Keywords:** methanol, formaldehyde, *Methylobacterium* species, chemotaxis, methyl-accepting chemotaxis protein

## Abstract

Motile bacteria take a competitive advantage in colonization of plant surfaces to establish beneficial associations that eventually support plant health. Plant exudates serve not only as primary growth substrates for bacteria but also as bacterial chemotaxis attractants. A number of plant-derived compounds and corresponding chemotaxis sensors have been documented, however, the sensors for methanol, one of the major volatile compounds released by plants, have not been identified. *Methylobacterium* species are ubiquitous plant surface-symbiotic, methylotrophic bacteria. A plant-growth promoting bacterium, *M. aquaticum* strain 22A exhibits chemotaxis toward methanol (methylotaxis). Its genome encodes 52 methyl-accepting chemotaxis proteins (MCPs), among which we identified three MCPs (methylotaxis proteins, MtpA, MtpB, and MtpC) responsible for methylotaxis. The triple gene mutant of the MCPs exhibited no methylotaxis, slower gathering to plant tissues, and less efficient colonization on plants than the wild type, suggesting that the methylotaxis mediates initiation of plant-*Methylobacterium* symbiosis and engages in proliferation on plants. To examine how these MCPs are operating methylotaxis, we generated multiple gene knockouts of the MCPs, and Ca^2+^-dependent MxaFI and lanthanide (Ln^3+^)-dependent XoxF methanol dehydrogenases (MDHs), whose expression is regulated by the presence of Ln^3+^. MtpA was found to be a cytosolic sensor that conducts formaldehyde taxis (formtaxis), as well as methylotaxis when MDHs generate formaldehyde. MtpB contained a dCache domain and exhibited differential cellular localization in response to La^3+^. MtpB expression was induced by La^3+^, and its activity required XoxF1. MtpC exhibited typical cell pole localization, required MxaFI activity, and was regulated under MxbDM that is also required for MxaF expression. Strain 22A methylotaxis is realized by three independent MCPs, two of which monitor methanol oxidation by Ln^3+^-regulated MDHs, and one of which monitors the common methanol oxidation product, formaldehyde. We propose that methanol metabolism-linked chemotaxis is the key factor for the efficient colonization of *Methylobacterium* on plants.

## Introduction

Microbial plant colonizers recognize the existence of plants by sensing the chemicals released by plants to establish their symbiotic or pathogenic relationships (Scharf et al., 2016). Most motile bacteria have an array of sensors called methyl-accepting chemotaxis receptors (MCPs). MCPs and the cooperating Che system that transmits the signals from MCPs to the flagellar motor regulate the direction of flagellar rotation and enable attractant-directed swimming, defined as chemotaxis (Karmakar, 2021). The chemotaxis mechanism has been extensively studied using *Escherichia coli* as a model organism. The *E. coli* genome encodes five MCP genes, whose detailed function and their ligands have been studied well (Sourjik and Armitage, 2010). In contrast, plant-colonizing bacteria possess huge numbers of MCPs. For example, *Rhizobium* species have 15 to 30 MCPs, *Agrobacterium* species have 20 to 40 MCPs, and *Bradyrhizobium* species have 30 to 60 MCPs (Scharf et al., 2016). These receptors typically contain an N-terminal ligand-binding region and a C-terminal signaling region containing a methyl-accepting domain. The ligand-binding domains of the large majority (~88%) of bacterial MCPs have not been annotated (Lacal et al., 2010).

*Methylobacterium* species are ubiquitous methylotrophic colonizers on plant aerial surfaces (phyllosphere), and they can occupy 10 to 20% of total culturable bacteria on plant surfaces (Vorholt, 2012). Methanol released by plants as a byproduct of pectin degradation (Fall and Benson, 1996) offers a niche for methylotrophic bacteria. Plant-associated methylotrophic bacteria are also capable of synthesizing phytohormones that can affect plant growth, resulting in plant growth promotion (Dourado et al., 2015). Thus, *Methylobacterium* species are recognized as mutual symbionts for plants.

*Methylobacterium* species oxidize methanol mainly using two different methanol dehydrogenases (MDHs), MxaFI and XoxF. The former is a calcium (Ca^2+^)-dependent enzyme, whereas the latter was found to be a lanthanide (Ln^3+^)-dependent enzyme (Anderson et al., 1990; Hibi et al., 2011; Nakagawa et al., 2012; Keltjens et al., 2014). Another Ln^3+^-dependent alcohol dehydrogenase, ExaF, was found in *Methylorubrum extorquens* strain AM1 (Good et al., 2016) and *Methylobacterium aquaticum* strain 22A (Yanpirat et al., 2020). Recognition and transport of Ln^3+^, Ln^3+^-dependent regulation of methylotrophy genes, and catalytic and regulatory activity of XoxF are currently emerging fields of study on methylotrophic bacteria (Skovran et al., 2011; Vu et al., 2016; Ochsner et al., 2019; Roszczenko-Jasińska et al., 2020). Though Lns are included in “rare earth metals,” the concentration of Ln^3+^ in soils is not low and is almost equivalent to those of copper, cobalt, and zinc (Kamenopoulos et al., 2016). XoxF is more widespread in methylotrophic bacteria than MxaF and is believed to be closer to an ancient form of MDHs (Chistoserdova, 2019; Skovran et al., 2019). Thus, Ln^3+^ has a pivotal regulatory role in the methylotrophy and physiology of methylotrophic bacteria (Tani et al., 2021).

*Methylobacterium aquaticum* strain 22A is an isolate from a hydroponic culture of a moss, *Racomitrium japonicum*, and is also a potent plant growth promoter (Tani et al., 2012). The strain has the MDHs described above (MxaFI and XoxF1) as well as ExaF (Tani et al., 2015; Masuda et al., 2018; Yanpirat et al., 2020). XoxF1 and ExaF are induced by Ln^3+^, whereas MxaF is induced in the absence of Ln^3+^. For formaldehyde oxidation, the strain employs two different pathways, the H_4_MPT pathway and the GSH pathway (Yanpirat et al., 2020).

The 22A genome encodes as many as 52 MCPs. As reported previously, we found that some bacteria including *Methylobacterium* species exhibit chemotaxis toward methanol (Tola et al., 2019). In this study, we revealed that strain 22A has three MCPs each responsible for chemotaxis towards methanol and its metabolite formaldehyde (here we define methylotaxis and formtaxis, respectively), the methylotaxis is realized by the coordination of MCPs with the methanol metabolism that is regulated by Ln^3+^, and the methylotaxis has a critical role in locating plants.

## Results

### Identification of three MCPs involved in methylotaxis

A microscopy-aided capillary-plug assay was employed to assess chemotaxis activity. Strain 22A methylotaxis was inducible by methanol (Supplementary Figure S1). Strain 22A cells were incubated in HEPES buffer (20 mM, pH 7.0) at 20°C for 2 h after overnight cultivation on methanol (as optimized in Supplementary Figures S1B,C). Strain 22A exhibited taxis toward DL-malate, DL-glycerate, and L-glutamate (Supplementary Figure S1D). The amino acid sequences of 52 MCP genes encoded in the strain 22A genome conserve a common MCP signal domain at their C-terminus in most cases, and other different domains, such as dCache, 4HB_MCP_1, and HAMP domains (Supplementary Figure S2). We generated single-gene knockout mutants for 15 genes that are induced by methanol in the transcriptome data of strain 22A (Masuda et al., 2018), but none of the mutants lost methylotaxis completely (Supplementary Figure S3). Because *mtpA* knockout strain (maq22A_c14925, Δ*mtpA*) exhibited significantly weaker methylotaxis than the wild type, we hypothesized that strain 22A has multiple methylotaxis MCPs. Then, we generated multi-gene knockouts based on Δ*mtpA* mutant. The additional knockouts of *mtpB* (maq22A_1p32440) and *mtpC* (maq22A_c15300) that also showed weaker methylotaxis in the screening, resulted in a complete loss of methylotaxis. Hereafter we call Δ*mtpA*Δ*mtpB*Δ*mtpC* mutant the TM (triple gene mutant).

**Figure 1:**
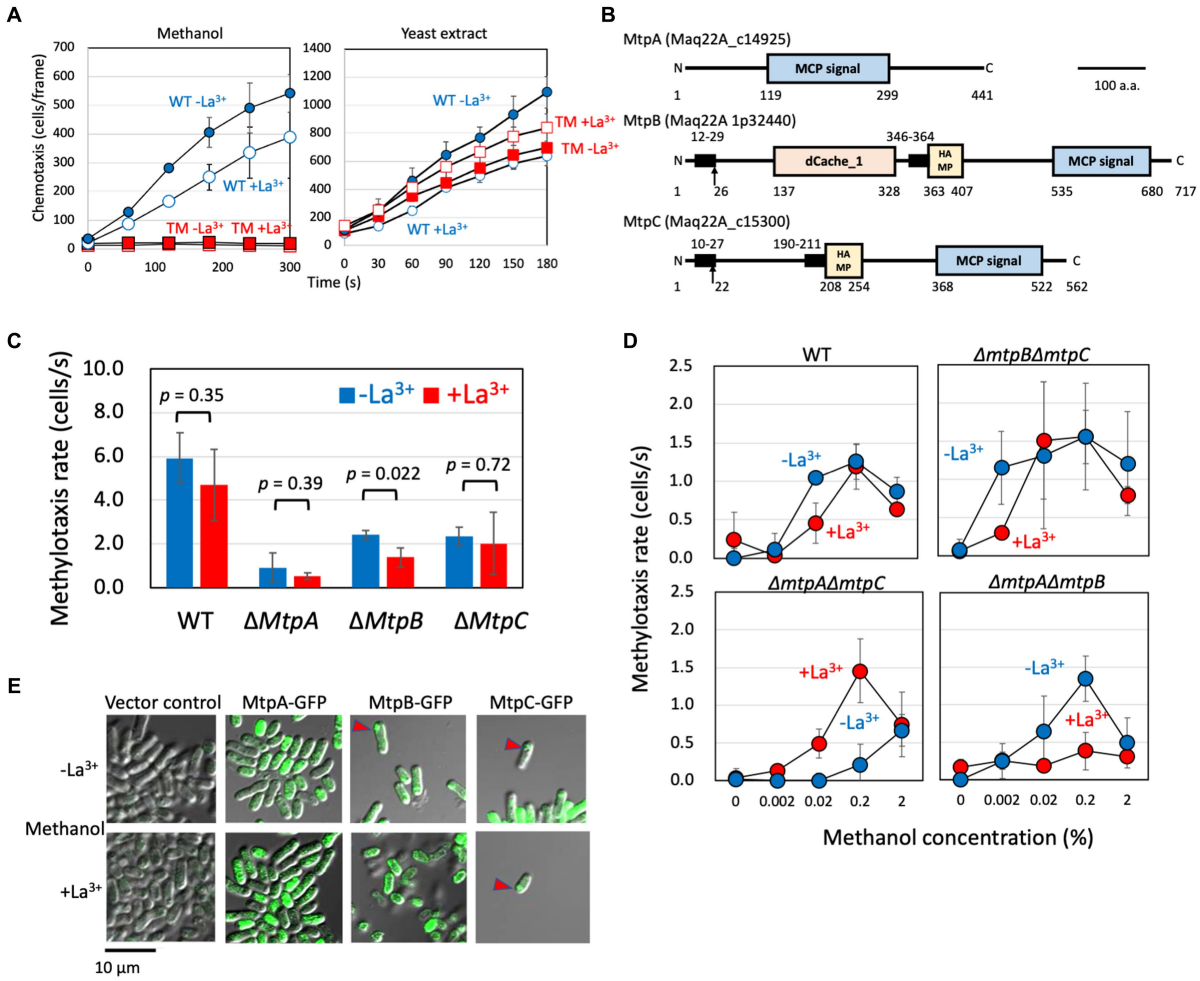
**(A)** Methylotaxis and chemotaxis toward the yeast extract of strain 22A wild type and TM grown on methanol in the absence and presence of LaCl_3_. The data are presented as the mean ± standard deviation (SD; *n* = 3). Blue (circle), wild type; red (square), TM; closed symbols, the absence of LaCl_3_; open symbols, the presence of LaCl_3_. The data are presented as the mean ± SD (*n* = 3). **(B)** Schematic diagram of methylotaxis MCPs structures. The conserved motifs, transmembrane domain, and signal peptide in the methylotaxis MCPs were analyzed by GenomeNet MOTIF Search (https://www.genome.jp/tools/motif/), TMHMM Server v 2.0 (http://www.cbs.dtu.dk/services/TMHMM/), and SignalP (http://www.cbs.dtu.dk/services/SignalP/), respectively. The motif search was carried out with the Pfam database, and a cut-off score e-value of 0.001. The transmembrane domain was regarded as the amino acids of score > 0.6. Motifs: MCP signal, PF00015 Methyl-accepting chemotaxis protein (MCP) signaling domain; HAMP, PF00672 HAMP domain; and dCache_1, PF02743 Cache domain (double cache; CAlcium channels and CHEmotaxis receptors). Arrows indicate putative signal peptide cleavage sites. Numbers indicate amino acid positions. Bar, 100 amino acids. **(C)** Methylotaxis rate of strain 22A wild type (WT) and single methylotaxis MCP gene knockouts, grown on methanol in the absence/presence of LaCl_3_. The data were analyzed with the Student’s *t*-test and are shown as the mean rate ± standard deviation (SD; *n* = 3). **(D)** Methylotaxis of strain 22A wild type (WT) and double MCP gene knockouts, grown on methanol in the absence/presence of LaCl_3_, toward varied concentrations of methanol. The data are shown as the mean rate ± SD (*n* = 3). **(E)** Cellular localization of GFP-tagged MCPs. The strain 22A wild type transformed with GFP-tagged MCP genes was grown on methanol in the presence/absence of LaCl_3_ for 2 days and subjected to confocal microscopy. Bar, 10 μm.

The TM grown on methanol in the presence and absence of LaCl_3_ did not exhibit any methylotaxis (Figure 1A) but retained chemotaxis toward yeast extract (2%) used as a mixture of amino acids. These results suggested that the mutant is not impaired in its chemotaxis machinery but only in attractant recognition and that the taxis response of La^3+^ is specific to methanol.

In MtpA amino acid sequence we detected only an MCP signaling domain (Figure 1B) involved in its dimerization and interaction with CheA protein and the adaptor protein CheW. The absence of the transmembrane region suggested that MtpA operates in the cytoplasm. MtpB contains a putative signal peptide, dCache_1 domain, a transmembrane domain, and a HAMP domain. The dCache_1 is the predominant sensor domain that recognizes wide variety of compounds including proteinogenic amino acids, polyamines, quaternary amines, purines, organic acids, sugars, quorum sensing signals, or inorganic ions (Matilla et al., 2021). The HAMP domain transmits the outside signal into the cytosol (Parkinson, 2010). MtpC contains a signal peptide, a transmembrane region, and a HAMP domain, exhibiting a typical structure of general MCPs. The predicted periplasmic domain has no sign for any known domains. Thus, we concluded that MtpA has atypical topology IV (cytosolic receptor), and MtpB and MtpC have typical topology I with an extracellular ligand binding domain, according to the classification by Lacal et al. (2010).

The single-gene knockouts of three MCPs exhibited different intensities of methylotaxis (Figure 1C). Deletion of *mtpA* resulted in a significant decrease in methylotaxis activity, suggesting its major contribution to cellular methylotaxis. Whereas methylotaxis of ∆*mtpA* and ∆*mtpC* mutants was not affected by the presence of LaCl_3_, Δ*mtpB* mutant exhibited weaker methylotaxis when it is grown on methanol in the presence of LaCl_3_ (hereafter called the MeOH+La condition) than when it is grown on methanol in the absence of LaCl_3_ (MeOH-La condition), suggesting that MtpB contributes to methylotaxis more in the presence of La^3+^. Indeed, *mtpB* expression is approximately twofold increased by La^3+^ (Supplementary Figure S3).

Then we examined the sensitivity and Ln-dependency of the methylotaxis of the double gene knockouts (Figure 1D), and found that MtpA is a relatively sensitive sensor, MtpB is a relatively less-sensitive sensor operating in the presence of La^3+^, and MtpC is a relatively less-sensitive sensor operating in the absence of La^3+^. These data indicated that each MCP with different sensitivity operates independently, and their expression and activity depend on the availability of La^3+^.

The methylotaxis MCPs were expressed as green fluorescent protein (GFP)(C-terminal)-fusion proteins under the control of their promoters in strain 22A wild type, and their cellular localization was examined with confocal microscopy (Figure 1E). The promoter-less vector control did not exhibit any fluorescence. MtpA-GFP localization was observed in the cytoplasm, as predicted above. The MtpB-GFP signal was observed at the pole of the cells grown in the MeOH-La condition, whereas interestingly, it was observed at the cell periphery in the MeOH+La condition. The MtpC-GFP signal was observed at the cell pole. It is known that essentially all of the MCP molecules cluster together with CheA and CheW to form the chemotaxis sensory array at the cell pole (Briegel et al., 2009).

The methylotaxis MCP genes with their promoter region were PCR-amplified and cloned into pAT01 (Fujitani et al., 2022) to generate pAT01-MtpA, -MtpB, and-MtpC. The methylotaxis of TM transformed with these plasmids was examined (Supplementary Figure S4). The TM carrying pAT01-MtpA showed comparable methylotaxis activity irrespective to the presence of LaCl_3_. The TM carrying pAT01-MtpB and-MtpC showed higher methylotaxis when grown in the presence and absence of LaCl_3_, respectively. These responses to La^3+^ were in line with the result obtained in Figure 1D. These gene complementation experiments indicated that the phenotype of the mutants was caused by the MCP gene deletion and not by polar effect.

The TM exhibited no growth defect on methanol, irrespective of the presence of La^3+^ (Supplementary Figure S5), suggesting that the methylotaxis MCPs are not involved in methanol metabolism and its regulation.

### Methylotaxis engages in locating plants and phyllospheric growth

To examine whether methylotaxis is involved in locating plants, first, we quantified the methanol exuded by rice roots in hydroponic culture (Supplementary Figure S6). After 8 h of seedling transplantation, the methanol concentration in the medium reached a maximum (3.5 mM = 0.01% w/v) and then gradually decreased. The methanol exudation rate was calculated to be approximately 0.113 mmol (=3.6 mg)/g plant fresh weight/h.

**Figure 2:**
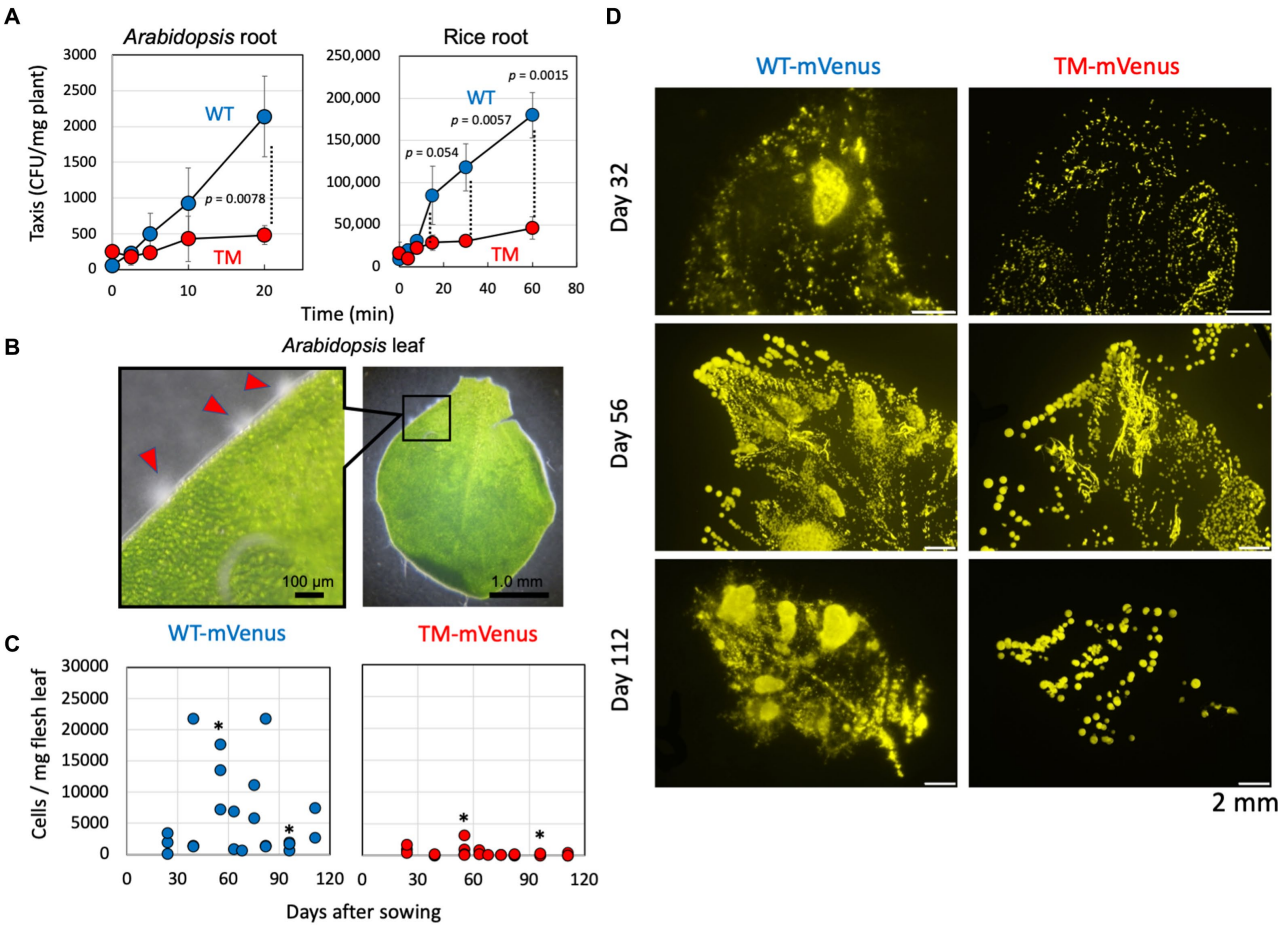
**(A)** Taxis of strain 22A wild type (WT) and TM for *Arabidopsis* and rice root. The taxis was evaluated as the CFUs attached to the plant root per fresh weight of the tissues. The data are presented as the mean ± standard deviation (*n* = 3) and analyzed with the Student’s *t*-test. **(B)** Gathering of strain 22A wild type cells to specific sites at the edge of young *Arabidopsis* leaf. Scales are shown in the pictures. **(C)** Quantification of mVenus-labeled strain 22A cells on the red perilla leaves. Cells of strains WT-mVenus and TM-mVenus were inoculated on red perilla seeds and the plants were cultivated. Fresh leaves were collected on the indicated days after sowing and mVenus-labeled cells were counted by flow cytometry. One to four leaves were sampled at each time point. Asterisks indicate *p* values less than 0.05 (Student’s *t*-test). **(D)** Fluorescence stereo-microscopic images of leaf-printed colonies of WT-mVenus and TM-mVenus. Fresh leaves collected 32, 56, and 112 days after sowing were impressed onto the methanol-agar plate, and colonies were observed by fluorescence stereo-microscopy. Bars indicate 2 mm.

The taxis toward *Arabidopsis* and rice roots was examined by counting the number of cells gathered and attached to the roots after soaking the roots in the cell suspensions (Figure 2A). The wild-type cells gathered to plant roots rapidly, but the TM exhibited less efficient gathering in the tested duration. These results suggested that the methanol and methylotaxis of strain 22A engage in locating the plants. The strain 22A wild-type cells gather at specific sites at the edge of *Arabidopsis* leaves, where they swim around actively (Figure 2B). These sites should be stomata or hydathodes, which are most possibly the sites releasing methanol.

To reveal the physiological role of methylotaxis in the phyllosphere, the ability of seed-inoculated strain 22A and TM to colonize plant leaves was compared. The cells were labeled with the fluorescent protein mVenus and inoculated on sterilized red perilla seeds that were cultivated aseptically. During cultivation, the cells washed off from the leaves were quantified by flow cytometry. Whereas the cell number of TM remained low during the course of experiment, at certain time points (e.g., at 55 and 96 days) the number of wild-type cells was significantly higher than that of TM cells (Figure 2C). These large fluctuations might be due to plasmid loss in the non-selective experimental setup and also to ununiform colonization pattern of the cells on individual plants and leaves. Leaf-imprinting also indicated that the wild type 22A colonized red perilla leaves more widely and densely than the TM (Figure 2D). These results suggested that methylotaxis contributes to efficient colonization in the phyllosphere.

### Formtaxis as a metabolism-linked methylotaxis

**Figure 3:**
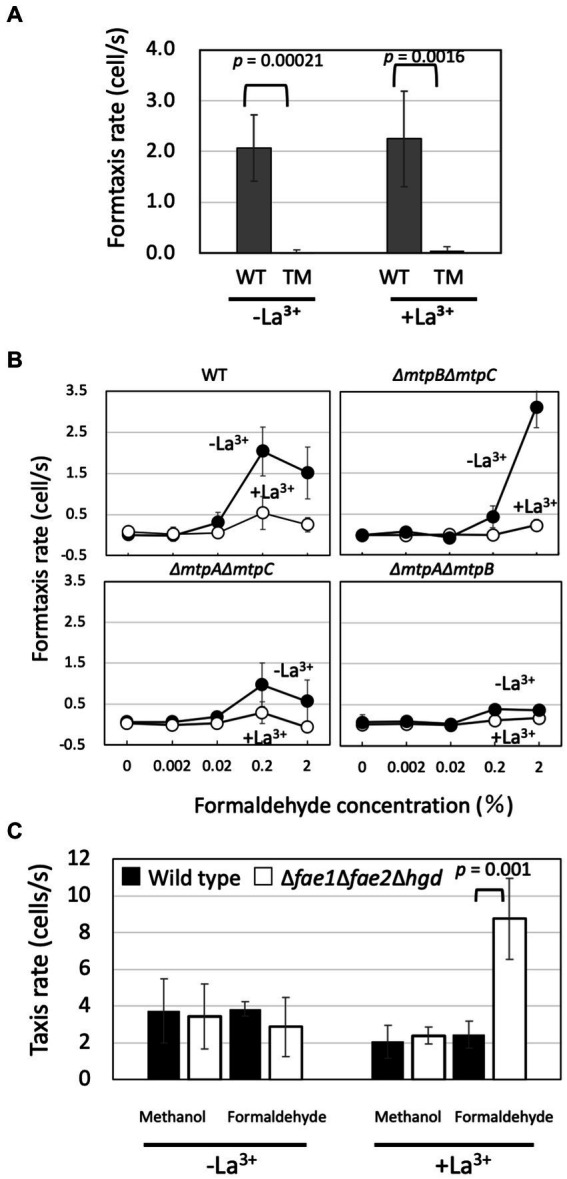
**(A)** Chemotaxis toward 0.2% formaldehyde (formtaxis) in strain 22A wild type (WT) and TM grown on methanol in the presence/absence of La^3+^. The data were analyzed with Student’s *t*-test and are shown as the mean rate ± standard deviation (SD; *n* = 5). **(B)** Formtaxis rate of strain 22A wild type and double methylotaxis MCP gene knockouts, grown on methanol in the absence/presence of LaCl_3_, toward varied concentrations of formaldehyde. The data are shown as the mean rate ± SD (*n* = 3). **(C)** Methylotaxis and formtaxis rates of the wild type and formaldehyde oxidation-deficient mutant (Δ*fae1*Δ*fae2*Δ*hgd*). Both strains were grown on succinate in the presence/absence of La^3+^ and subjected to taxis assay. The data were analyzed with the Student’s *t*-test and only the significant *p*-value (*p* < 0.05) is indicated. The data are shown as the mean rate ± SD (*n* = 3).

*Methylobacterium* species oxidize methanol rapidly with MDHs, and the product formaldehyde is taken into the cytosol for further oxidation and assimilation. However, it is also released extracellularly (Masuda et al., 2018) from the periplasm where the oxidation by MDHs occurs. If strain 22A is chemotactic to formaldehyde, the methylotaxis observed above may include formtaxis. Strain 22A exhibited formtaxis irrespective of La^3+^ and the TM did not exhibit any formtaxis (Figure 3A). This result suggested that any of the methylotaxis MCP(s) is also involved in formtaxis, and no MCPs other than these three MCPs encoded in the genome are involved in formtaxis, as long as the strain is grown in the used conditions.

Next, we examined the formtaxis in double-gene knockouts of methylotaxis MCPs with varied concentrations of formaldehyde (Figure 3B). The wild type exhibited stronger formtaxis when grown in the MeOH-La condition than in the MeOH+La condition, and the best concentration of formaldehyde was 0.2%. Δ*mtpB*Δ*mtpC* mutant exhibited strong formtaxis compared to the wild type, for the high concentration of formaldehyde (2%) only when it was grown in the MeOH-La condition. Δ*mtpA*Δ*mtpC* mutant exhibited moderate formtaxis when grown in the MeOH-La condition, for 0.2% formaldehyde. Δ*mtpA*Δ*mtpB* mutant exhibited almost no formtaxis at any tested concentrations of formaldehyde. Thus, MtpC is involved only in methylotaxis and not in formtaxis.

Strain 22A did not exhibit any taxis toward 2 and 0.2% formate that is also a metabolite in methylotrophic pathway (data not shown), although formate serves as a chemoattractant for a soil plant pathogen, *Agrobacterium fabrum* strain C58 (Wang et al., 2021).

The strain 22A genome encodes genes for the H_4_MPT pathway that plays a central role in formaldehyde oxidation, starting with the reaction catalyzed by the formaldehyde-activating enzyme (Fae). Strain 22A has two homologous *fae* genes (*fae1* and *fae2*). Strain 22A (and many other *Methylobacterium* strains in the C1 clade, Alessa et al., 2021) have additional glutathione-dependent formaldehyde oxidation pathway (GSH pathway) genes composed of *gfa* (glutathione-dependent formaldehyde-activating enzyme), *hgd* (S-hydroxymethyl glutathione dehydrogenase), and *fgh* (S-formylglutathione hydrolase). The Δ*fae1*Δ*fae2*Δ*hgd* mutant is completely defective in formaldehyde metabolism and unable to grow even on succinate in the presence of methanol, due to formaldehyde toxicity (Yanpirat et al., 2020). The mutant grown in the absence of methanol did not lose methylotaxis and formtaxis (Figure 3C), suggesting that methylotaxis and formtaxis do not require the formaldehyde metabolic pathways. Whereas the chemotactic cells usually gather and swim around the capillary mouth in the assay, the mutant cells could no longer swim once gathered around the capillary, suggesting that they were toxified by formaldehyde (data not shown). The Δ*fae1*Δ*fae2*Δ*hgd* mutant exhibited stronger formtaxis than the wild type in the presence of La^3+^, the possible reason for which is discussed below.

### Examination of the relationships between MDHs and MCPs revealed that MtpA is responsible for formtaxis

To examine the involvement of MDHs in methylotaxis and formtaxis, we tested MDH gene knockouts of strain 22A grown on succinate plus methanol. XoxF1 is necessary for the expression of MxaF, therefore, Δ*xoxF1* mutant cannot grow on methanol, irrespective of La^3+^. Δ*mxaF* mutant can grow on methanol only in the presence of La^3+^ due to intact XoxF1. Δ*xoxF1*Sup mutant is a suppression mutant derived from Δ*xoxF1* mutant that carries a mutation in *mxbD* encoding a sensor kinase responsible for *mxaF* expression, and the mutant restored the *mxaF*-dependent growth on methanol without *xoxF1* (Yanpirat et al., 2020).

**Figure 4:**
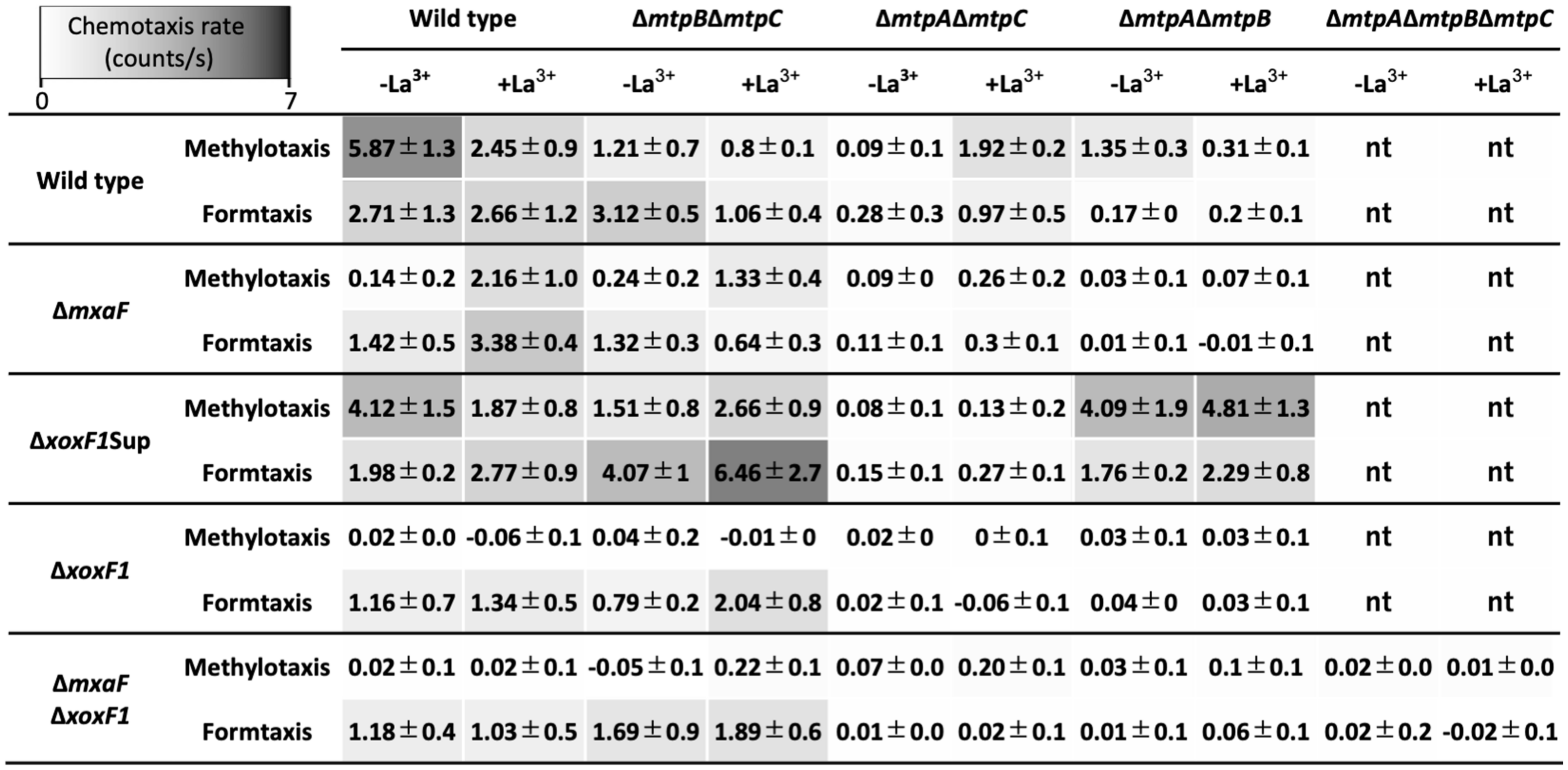
Methylotaxis and formtaxis rates of a series of MCP gene knockouts generated under the backgrounds of various MDH gene knockouts. Data are presented as the mean chemotaxis rate ± standard deviation (*n* = 3). nt, not tested.

Δ*xoxF1* and Δ*xoxF1*Δ*mxaF* mutants did not exhibit any methylotaxis but retained formtaxis (Figure 4). *∆mxaF* mutant grown in the presence of La^3+^ and ∆*xoxF1*Sup retained methylotaxis. Thus, either of the active MDHs is necessary for methylotaxis, and formtaxis does not necessitate active MDHs. Namely, there may be MCP(s) that recognize(s) formaldehyde.

To differentiate which of methanol and formaldehyde is recognized by each MCP, we generated double methylotaxis MCP gene knockouts in the background of a series of MDH gene knockouts (Figure 4). All mutants were motile under microscopic observation, and TM under ∆*mxaF*∆*xoxF1* background exhibited wild-type level of taxis toward yeast extract (data not shown), supporting the idea that the defect in methylotaxis was not due to the loss of swimming motility.

The Δ*mtpB*Δ*mtpC* knockout mutants under Δ*xoxF1* and Δ*mxaF*Δ*xoxF1* backgrounds retained formtaxis irrespective of La^3+^, whereas they lost methylotaxis, suggesting that MtpA-dependent methylotaxis depends on formaldehyde generation by MDHs, and MtpA-dependent formtaxis does not require MDHs. Therefore, MtpA is responsible for formtaxis. The relatively high formtaxis in Δ*mtpB*Δ*mtpC* mutant under Δ*mxaF*Δ*xoxF1* background in the presence of La^3+^ might be due to formaldehyde generation by ExaF that is active for methanol as well as ethanol. Interestingly, the Δ*mtpB*Δ*mtpC* mutant under Δ*xoxF1*Sup background exhibited high methylotaxis and formtaxis. Δ*xoxF1*Sup mutant constitutively and highly expresses *mxaF*. Therefore, formaldehyde generated by MxaF might promote the taxis.

The vector for the MtpA-GFP construct was introduced into the TM, and the transformant exhibited formtaxis (Supplementary Figure S7), suggesting that the cytosol-localized GFP-tagged MtpA (Figure 1E) is functional.

### MtpB depends on MDH activity and La^3+^

The Δ*mtpA*Δ*mtpC* mutants under any MDH knockouts lost methylotaxis and formtaxis in the absence of La^3+^ (Figure 4), partly because the expression of *mtpB* is low in the absence of La^3+^(Supplementary Figure S3). In the presence of La^3+^, the Δ*mtpA*Δ*mtpC* mutant under Δ*mxaF* and Δ*xoxF1*Sup backgrounds retained low but significant levels of taxis, because either of the MDHs is operating in these mutants.

The strong formtaxis exhibited in Δ*fae1*Δ*fae2*Δ*hgd* mutant in the presence of La^3+^ (Figure 3C) might be due to the formaldehyde accumulation caused by the mutation and the participation of the MtpB activity sensing XoxF1-dependent formaldehyde oxidation.

Strain 22A grown on methanol also exhibited ethanol-taxis, and the assay with double MCP gene knockouts suggested that MtpB contributes most to the ethanol-taxis (Supplementary Figure S8A). TM exhibited weak activity in the absence of La^3+^, suggesting that strain 22A has other unidentified MCPs for ethanol-taxis that operate, especially in the absence of La^3+^. The methylotaxis activity was competitively lost in the presence of high concentrations of ethanol (Supplementary Figure S8B).

### MtpC activity necessitates MxaF and is under the regulation of MxbDM

**Figure 5:**
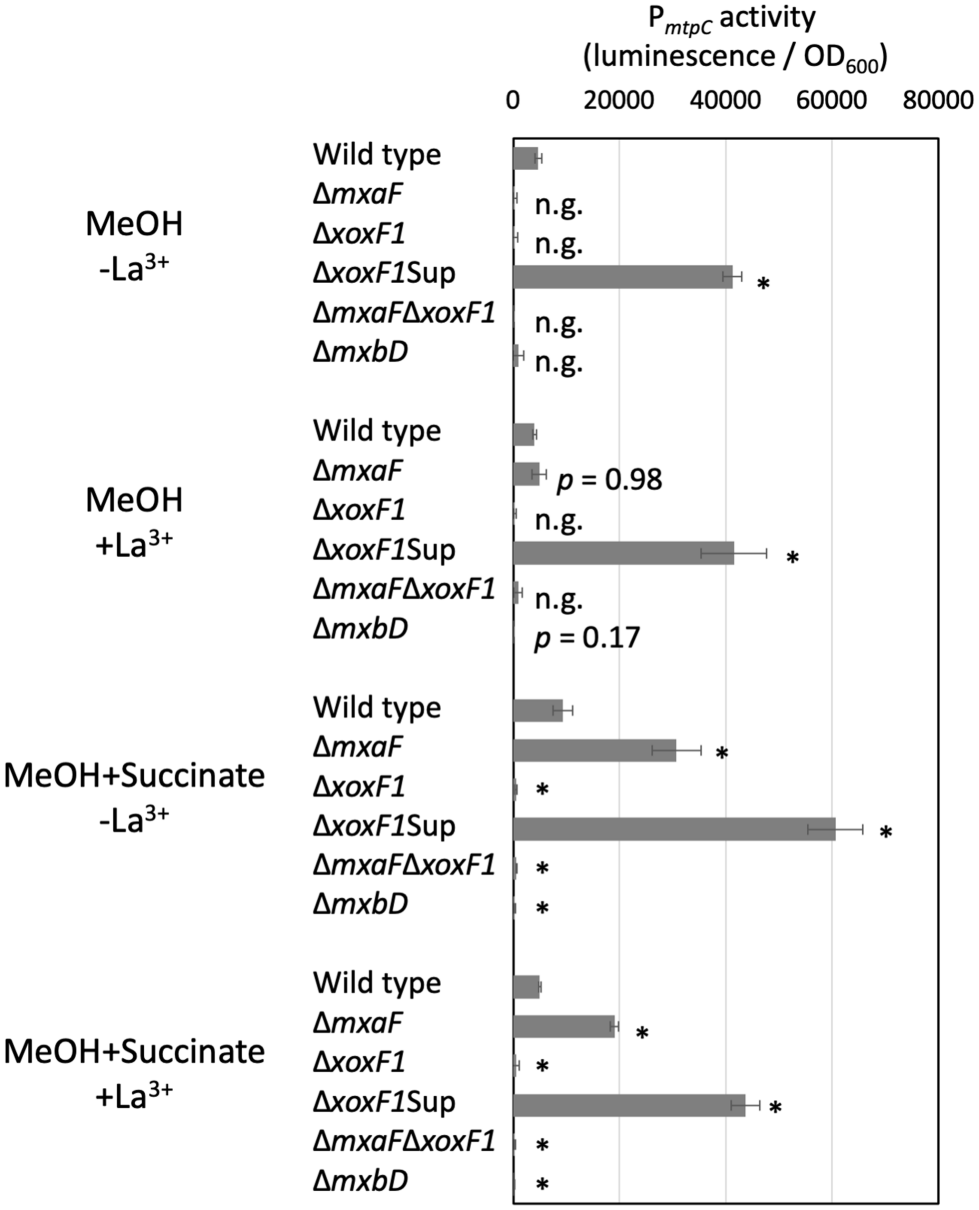
P*_mtpC_* activity in various MDH and ∆*mxbD* mutants. Strain 22A MDH knockouts and *∆mxbD* mutant carrying pAT06-P*_mtpC_* were grown on methanol, succinate, or methanol plus succinate in the absence/presence of La^3+^ in liquid medium prepared in 96-well plates. The data were analyzed with analysis of variance followed by Dunnet’s test to compare the differences against wild-type data. *p* values lower than 0.01 are indicated by asterisks. The data are shown as mean luminescence/OD_600_ ± SD (*n* = 4). n.g., no growth.

The Δ*mtpA*Δ*mtpB* mutants exhibited almost no methylotaxis or formtaxis under backgrounds of Δ*mxaF*, Δ*xoxF1*, and Δ*mxaF*Δ*xoxF1*, but exhibited strong taxis for both attractants under a Δ*xoxF1*Sup background (Figure 4), suggesting that the activity or expression of MtpC depends on MxaF. To examine whether the expression of MtpC depends on the expression of *mxaF*, we measured the promoter activity of *mtpC* (P*_mtpC_*) in various MDH knockouts using a promoter-reporter (luciferase) vector (Figure 5). The P*_mtpC_* activity was notably higher in Δ*xoxF1*Sup mutant, and negligible in Δ*xoxF1* and Δ*mxaF*Δ*xoxF1* mutants. The P*_mtpC_* activity was not lost in Δ*mxaF* mutant, suggesting that the expression of *mtpC* is not dependent on the presence of *mxaF*. Therefore, we conclude that MtpC is dependent on MxaF activity but not on its expression. Namely, the ligand for MtpC is not methanol.

*mxaF* expression is dependent on the presence of *xoxF1* and a two-component signaling system, MxbDM. The P*_mtpC_* activity was abolished almost completely in the ∆*mxbD* mutant, suggesting that *mtpC* expression is under regulation by *mxbDM*, which also regulates *mxaF*.

## Discussion

Chemotaxis is one of the bacterial survival strategies that enable the cells to reach nutrients, and also to establish symbiotic and associative relationships between hosts and microorganisms (Allard-Massicotte et al., 2016; Scharf et al., 2016; Feng et al., 2019). Previously, we showed that environmental bacteria including *Methylobacterium* species exhibited methylotaxis (Tola et al., 2019). However, its molecular mechanism and contribution to bacterial survival have remained elusive. In this study, we proved that a methylotrophic plant–growth promoting bacterium *M. aquaticum* strain 22A has as many as three MCPs for methylotaxis (Figure 1).

Strain 22A utilizes methylotaxis to locate the plant and to initiate symbiosis, and gathers in specific niches of natural openings, such as stomata and hydathodes (Figures 2A,B). The seed-inoculated TM exhibited less efficient colonization on perilla leaves than the wild type (Figures 2C,D). It is currently unknown whether this is a result of the fewer opportunities to reach favorable niches that eventually support bacterial cell proliferation or the cellular spontaneous migration from seeds to leaves on the plant surface or through internal tissues of the vascular system (Chi et al., 2005; Ji et al., 2010). Methylotaxis is one of the survival strategies that enable *Methylobacterium* cells to reach nutrients and to establish symbiosis with plants.

Although three MCPs are involved in the same cellular function of methylotaxis, they operate independently and have distinctly different characteristics in their secondary structures, induction response to La^3+^, sensitivity, and cellular localization. To add an evolutionary viewpoint to their function and conservation, we examined MCP genes in the genomes of 62 type strains of the genera (1,467 MCP genes in total, 23.3 genes per genome, Supplementary Table S1). The type strains of clade A and C with relatively larger genomic sizes contain relatively larger numbers of MCP genes (average: 34 genes). We also tested the methylotaxis activity of the type strains grown in the MeOH+La condition that supports the growth of all type strains. Twenty-six strains among the tested 60 type strains exhibited swimming motility, and all of them exhibited methylotaxis with different intensities. The methylotaxis MCP genes found in this study were not necessarily conserved in these strains, suggesting that another type of methylotaxis MCP exists.

MtpA was found to function in the cytosol (Figure 1E; Supplementary Figure S7) and is primarily responsible for formtaxis (Figure 3B), but also engages in methylotaxis when MDH is functional (Figure 4). MtpA is considered to be a sensor for methylotaxis that navigates the cells to the place where more formaldehyde is produced. Although formaldehyde can also be detected in plants (Blunden et al., 1998), considering the difference in sensitivity of methylotaxis and formtaxis (Figures 1D, 3B), abundant methanol emission from plants (Supplementary Figure S6), and ubiquity of functional MDHs in *Methylobacterium* species, methylotaxis would first be prioritized to locate plants in nature. It is reported that *E. coli* cells are supposed to secrete serine upon being attracted by aspartic acid, and serine is used as a signaling molecule to attract other cells (Long et al., 2017). The formaldehyde secreted by the methanol-oxidizing cells may serve as a secondary signal to gather other *Methylobacterium* cells and to enhance the colonization of the species. Although MtpA is conserved in clade C members (Supplementary Table S1) in which the GSH pathway is also conserved (Alessa et al., 2021), the formtaxis did not necessitate the formaldehyde metabolism pathways. Thus, it is unlikely that a metabolic intermediate (such as S-formylglutathione, H_4_MPT, etc.) in the pathways is the ligand for MtpA. The clade C members are considered to be evolutionary ancestors that may prefer soil, rhizosphere, or aquatic environments to the phyllosphere (Leducq et al., 2022). It is interesting to hypothesize that MtpA-dependent formtaxis may contribute to colonizing a niche by convening colleagues in such environments. There is no hint for ligand based on MtpA structural information. It is reported that about one-quarter of bacteria cytoplasmic chemoreceptors had no identifiable domain, whereas about half of analyzed chemoreceptors contain PAS domain that is involved mainly in energy taxis (Collins et al., 2014). Identification of the ligand for MtpA is currently underway.

MtpB was found to depend on MDH activity and La^3+^ (Figure 4). Its cellular localization changed in response to La^3+^ (Figure 1E), which is unique, as well as reasonable to detect the MDH activity that operate in the periplasm. The molecular mechanism that regulates and alters the protein localization is unknown. As one of the examples of differential localization of MCP protein, the *Azospirillum brasilense* aerotaxis sensor AerC exhibits different localization: the cell pole in the nitrogen fixation condition and the cytosol in the presence of ammonium (Xie et al., 2010). The dCache_1 domain generally recognizes various compounds, and majority of known ligands are amino acids (Upadhyay et al., 2016). Because its activity depends on MDH activity, the ligand for MtpB is not methanol itself. MDH genes (*mxaFI* and *xoxF1*) are associated with solute-binding protein genes (*mxaJ* and *xoxJ*) in each cluster. *mxaJ* deletion in *M. extorquens* AM1 resulted in disruption of MxaFI activation (Amaratunga et al., 1997). MxaJ is suggested to be crucial for MxaFI maturation, however, its exact role is unknown. On the other hand, solute binding proteins are known to function as ligands for many different types of signal transduction receptors, including MCPs that contain dCache, HBM or TarH domains (Matilla et al., 2021). Therefore, it is possible that the ligand for MtpB is XoxJ. MtpB is also involved in formtaxis and ethanol-taxis (Supplementary Figure S8) due to the formaldehyde and ethanol oxidation capacity of XoxF1. Ethanol is released from plants (Fall, 1999), and is also a good growth substrate for strain 22A (Yanpirat et al., 2020). Most probably, ethanol-taxis is dependent on MtpB and XoxF1 or ExaF activity. As we reported previously, Ln^3+^-dependent methylotrophy is completely conserved in the genera whereas some strains lack the *mxa* cluster (Alessa et al., 2021), and XoxF-type MDH is considered to be ancestral to MxaF-type MDH. Therefore, it is reasonable that MtpB monitoring XoxF1 activity is conserved widely in the genera (Supplementary Table S1). MtpB is relatively less conserved in clade D members that have relatively smaller genomes as well as smaller numbers of MCPs. Clade D contains specialized forest tree phyllospheric members (Leducq et al., 2022) that may have limited access to Ln^3+^.

**Figure 6:**
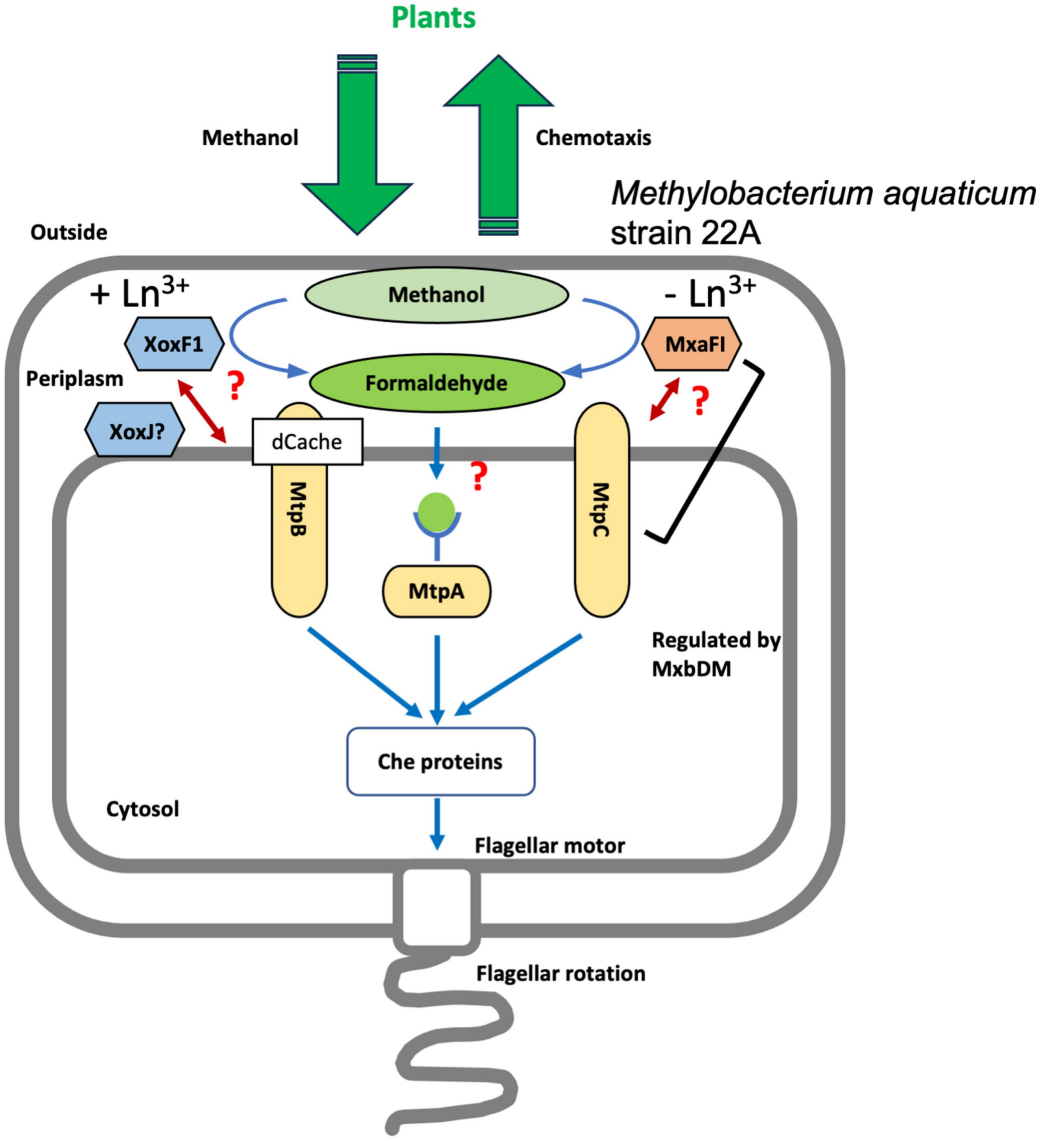
A schematic representation of the putative mechanism of strain 22A methylotaxis. Methanol released by plants is oxidized to formaldehyde in the periplasm by either MxaF (with its beta subunit, MxaI) or XoxF1 depending on the availability of Ln^3+^. MtpA localizes in the cytosol and presumably recognizes formaldehyde as a ligand. MtpB is induced in the presence of Ln^3+^, and its activity is coupled with XoxF activity. MtpC is induced in the absence of Ln^3+^, and its activity is coupled with MxaF activity directly or indirectly. The expression of MtpC and MxaF is regulated under the control of MxbDM. The signals were integrated and transmitted to Che proteins that control the direction of flagellar rotation, resulting in methylotaxis and successful cell gathering to plants.

MtpC was found to be associated with MxaF activity (Figure 4). Its expression is independent of *mxaF* expression but dependent on *mxbD*, which regulates *mxaF* expression in the absence of La^3+^ (Figure 6). Thus, *mxbD* regulates not only MxaF-dependent methylotrophy but also methylotaxis when Ln^3+^ is not available. As one of the similar regulators, ArcA from avian pathogenic *E. coli* also controls chemotaxis as well as metabolism and motility (Jiang et al., 2015). Because MtpC cellular localization did not change in response to La^3+^, and its amino acid sequence does not contain any known domains, its mode of action may differ from that of MtpB. MtpC will not bind methanol directly as a ligand, and it is unknown how MtpC activity cooperates with MxaF activity. MtpC appeared randomly across the type strain genomes but not in the strains that lack *mxa* genes (*M. tardum* NBRC 103632^T^, *M. longum* DSM 23933^T^, *M. persicinum* NBRC 103628^T^, *M. komagatae* DSM 19563^T^, and *M. iners* DSM 19015^T^; Supplementary Table S1, Alessa et al., 2021), implying the functional and evolutionary link between MtpC and MxaFI.

Thus, strain 22A is equipped with as many as three distinct MCPs that enable its methylotaxis with different mechanisms. They navigate the cells to the niche where methanol is more available by monitoring the cellular methanol oxidation and its product formaldehyde, depending on which MDH is operating in response to the availability of Ln^3+^. A schematic representation of possible methylotaxis mechanisms in strain 22A is provided in Figure 6.

Many kinds of plant-derived chemicals, including organic acids, amino acids, phenolics, and sugars, support plant-associated bacterial survival (Chaparro et al., 2013). They have also been identified as cues for bacteria chemotaxis to locate the plants (Feng et al., 2018). Within such a wide variety of chemotaxis, we propose that methylotaxis is one of the crucial steps for methylotrophs to initiate symbiosis. Further investigation of ligand identification for methylotaxis MCPs, the roles of many other sensors encoded in the strain 22A genome, and the identification of other types of methylotaxis sensors in the other *Methylobacterium* species will contribute to revealing the eco-physiological and evolutionary versatility of *Methylobacterium* species.

## Materials and methods

### Strains and culture conditions

*M. aquaticum* strain 22A (FERM-BP11078, Tani et al., 2012) was used for MCP identification. Other *Methylobacterium* type strains were also tested for methylotaxis. *Methylobacterium* strains were usually grown on R2A medium or mineral medium (MM; Alamgir et al., 2015) containing 0.5% methanol or 0.5% succinate, or both. Kanamycin (25 mg/L) and 1 μM LaCl_3_ were added when necessary. For growth experiments, strain 22A and its derivatives were grown on 200 μl medium prepared in 96-well plates, which were rotary-shaken at 300 rpm at 28°C. The growth was monitored by measuring optical density at 600 nm using a microplate reader (PowerScan HT, DS Pharma). MeOH-La and MeOH+La conditions refer to MM medium containing 0.5% methanol in the absence and presence of 1 μM LaCl_3_, respectively. *E. coli* DH5α was used for plasmid construction and *E. coli* S17-1 was used for conjugation, and they were grown on LB medium.

### Gene knockouts

Gene knockout mutants for methanol dehydrogenases, that is, Δ*mxaF*, Δ*xoxF1*, Δ*xoxF1*Sup and Δ*mxaF*Δ*xoxF1,* and *mxbD*, were generated in our previous studies (Masuda et al., 2018; Yanpirat et al., 2020). Gene knockout mutants for MCP genes were generated using an allele-replacement vector pK18mobSacB, as previously reported (Alamgir et al., 2015). In brief, the gene knockout vectors were constructed so that the vector contains tandem ligated each approximately 1 kb upstream and downstream regions of the gene to be deleted, using the primers listed in Supplementary Table S2 and the In-Fusion Cloning kit (Takara Bio Co.). The vector was introduced into strain 22A via conjugation using *E. coli* S17-1. Single-crossover mutants were selected by kanamycin resistance, and double-crossover mutants were selected by sucrose resistance. Polymerase chain reaction diagnosis was carried out as described previously (Alamgir et al., 2015).

### Chemotaxis assay

The cells grown on solid medium overnight were collected with a scraper, suspended in 2 to 3 ml of 10 mM HEPES pH 7.0 (OD_600_ = 0.01) in 15-ml tube, and incubated at 20°C for 2 h. Capillaries were made of a glass capillary (with filament, model GD-1, Narishige), with a glass puller (Narishige Model PC-10) so that the tip diameter becomes 10 to 20 μm. NuSieve GTG agarose (Lonza Bioscience, Co.) was dissolved in 10 mM HEPES pH 7.0 (15 mg/ml) at 50°C. Methanol (and other substances) was added at the desired concentration (2% v/v or w/v, otherwise stated) and the solution was kept at 50°C under seal. When formaldehyde was used as a chemoattractant, 35 mg paraformaldehyde was dissolved in 175 μl 10 mM HEPES pH 7.0 and incubated at 120°C for 15 min, and the solution was regarded as 20% (w/v) formaldehyde. The capillary tip was soaked in the solution for 5 s, and rinsed with water. A sheet of FastWell™ (2 mm thickness, 20 mm diameter, Grace Bio-Labs) was placed on a slide glass, and the 170 μl cell suspension was pipetted into the well. The density of the cell suspension was appropriately adjusted so that 50 to 100 cells are evident in the microscopic window. A cover glass was placed offset, allowing a capillary inserted between the FastWell and the cover glass. The bacteria gathering at the capillary tip were monitored with a light microscope (×200 magnification, Olympus, Tokyo, Japan) for 3 or 5 min, and pictured every 30 or 60 s (308 × 256 μm, 1,024 × 768 pixels) with a digital camera (VB-7010, Keyence). The cells in a frame were counted with ImageJ software (Schneider et al., 2012). Automatic cell counting was carried out with a macro containing the “Otsu” thresholding and “analyze particles” command. The assay was done in technical triplicates at least. The time-dependent gathering data were used to determine the intensity (gathering speed) of chemotaxis by measuring the slopes of the plots.

### Chemotaxis toward plant tissues

The gathering and adhesion of strain 22A cells to rice or *Arabidopsis thaliana* roots were assessed as follows in technical triplicate. *A. thaliana* col-0 seeds were surface-sterilized with 70% ethanol (2 min), 7% sodium hypochlorite containing 0.2% Triton X-100 (8 min), and thorough washing with sterile water. The seeds were allowed to germinate on 1/2 Murashige-Skoog (MS) medium in square plates (14 cm × 10 cm) solidified with 1.2% agar at 23°C, for 20 days (12 h light/dark cycle). The plates were set vertically to allow the roots to grow on the agar surface. The plants were removed, weighed, and rinsed with 10 mM HEPES pH 7.0. The root part only was soaked in 5 ml strain 22A cell suspension (OD_600_ = 0.01, 10 mM HEPES, pH 7.0, pre-grown in MeOH-La condition for 2 days) prepared in 6-well plates (1 plant/well). At appropriate intervals within 0 to 20 (or 60) min, the root was transferred into 1 ml HEPES buffer in 1.5 ml tubes and homogenized with a pestle. The homogenates were serially diluted with 10 mM HEPES pH 7.0, and 10 μl of the dilutes were spread onto solid MM containing 0.5% methanol. The bacterial colony forming units (CFUs) were determined after 5 days of incubation at 28°C.

The experiment using rice roots was carried out similarly. Rice seeds were dehulled, soaked in 70% ethanol for 3 min, and incubated in 3% sodium hypochlorite containing 0.5% tween 20 at 80°C for 30 min. The rice seeds (*Oryza sativa* cv. Nipponbare) were put on 0.8% agar and allowed to grow at 28°C for 10 days. The excised rice roots were incubated in bacterial cell suspension and transferred into 5 ml HEPES containing 0.05% Sylwet (BioMedical Science, Tokyo) and vigorously vortexed for 10 s to remove the cells.

The density (CFU/ml) of the cell suspension (5 ml) was wild type 2.5 ± 0.63 × 10^5^ and TM 3.2 ± 0.26 × 10^5^ in the *Arabidopsis* experiment, and wild type 2.0 ± 0.18 × 10^6^ and TM 1.4 ± 0.21 × 10^6^ in rice experiment.

A young leaf of *A. thaliana* grown as above was excised and put onto a slide glass, onto which strain 22A cell suspension was added. The bacterial cells were observed under a stereo microscope (Olympus MVX10).

### Quantification and observation of mVenus-labeled cells on red perilla leaves

The DNA fragment encoding mVenus protein was amplified by polymerase chain reaction using pDAS2V (Takeya et al., 2018) as a template and cloned into the EcoRI-digested pAT02m (Yanpirat et al., 2020) by the In-Fusion cloning kit. The plasmid, pAT02-V, was introduced into strains 22A and TM by electroporation (2 kV, 25 μF, 200 Ω) using a Gene Pulser Xcell (Bio-Rad Laboratories, Hercules, CA). Red perilla (*Perilla frutescens crispa* [Thunb.] Makino) seeds were sterilized with 70% ethanol for 1 min, and with 1% sodium hypochlorite (containing 0.3% Tween 20) for 1 min, then washed with sterilized water 5 times. Strains 22A and TM were cultivated in MM containing 0.5% methanol and 20 μg/ml kanamycin for 2 days at 28°C and cells were collected, washed with sterilized water, and suspended in sterilized water to obtain cell suspensions with OD_610_ of 0.1. The sterilized red perilla seeds were soaked in the cell suspension of strains 22A or TM for 3 h with gentle shaking at 2 rpm using a Rotator RT-5 (Taitec, Saitama, Japan) at room temperature. The treated seeds were sown onto Hoagland agar (culture dish, 100 mm × 40 mm, with porous film seal) and aseptically grown in a chamber (NK Biotron LH-220S, Nippon Medical and Chemical Instruments, Osaka, Japan) for 112 days. The system was operated at 25°C under a 16 h light and 8 h dark cycle.

Fresh leaves were removed from the perilla plants aseptically, put into a 1.5 ml tube, and weighed. Phosphate-buffered saline (PBS) was added to the tube (100 μl/10 mg leaf) and vigorously mixed with a Vortex mixer for 15 min. The 30 μl of suspension and 30 μl of AccuCheck Counting Beads (ThermoFisher Scientific, MA) were added to 300 μl of PBS, and mVenus-labeled cells were counted by flow cytometry (FACSAria IIIu, Becton Dickinson, San Jose, CA). For observation by fluorescence microscopy, one fresh leaf was impressed onto a solid MM containing 0.5% methanol and 20 μg/ml kanamycin for 1 min. After removal of the leaf, the plates were incubated for several days at 28°C and colonies were observed with a fluorescence stereo-microscope (Olympus SZ16) equipped with a digital charge-coupled device camera (Olympus DP80) and a YFP filter (Olympus SZX2-FYFPHQ).

### Promoter-reporter assay

The polymerase chain reaction–generated DNA of the *mtpC* promoter region (upstream non-coding region of the gene, 455 bp) was cloned into the *Nco*I site on pAT06-Lux that is a promoter-less bacterial luciferase reporter vector (Juma et al., 2022). The vector (pAT06-P_mtpC_) was introduced into strain 22A derivatives via conjugation using *E. coli* S17-1 and selected on kanamycin. The transformants were grown in 200 μl MM containing methanol or succinate or both, in the presence or absence of LaCl_3_. The culture OD_600_ and luminescence were measured with a microplate reader (PowerScan HT, DS Pharma). The promoter activity was regarded as the luminescence / OD_600_ when the luminescence reached a maximum during the cultivation.

### Cellular MCP localization analysis

To observe the cellular localization of MCPs, *mtpA* (maq22A_c14925), *mtpB* (maq22A_1p32440), and *mtpC* (maq22A_c15300) genes with their promoter region were cloned in tandem with the GFP gene into the *Pst*I site of pCM130KmC (Yanpirat et al., 2020) using the In-Fusion technique and the primers listed in Supplementary Table S2 to generate pCM130KmC-MtpA (or MtpB and MtpC)-GFP. The plasmids were transferred into strain 22A wild type or TM via conjugation by *E. coli* S17-1 and grown on 0.5% methanol in MM for 1 day. The cells were subjected to confocal microscopic observation (FV1000, Olympus). As a control, the GFP ORF fragment (without its promoter) was cloned into pCM130KmC.

### Gene complementation

The methylotaxis protein genes and their promoter regions were each PCR-amplified with the primers listed in Supplementary Table S2, and cloned into EcoRI site of pAT01 that enables His-tagged protein expression in *M. aquaticum* strain 22A (Fujitani et al., 2022). We introduced termination codon in the 3′ primers to avoid possible MCP activity interference by the tag. The vectors were each introduced into TM, and the transformants were grown on methanol in the presence and absence of LaCl_3_ and subjected to methylotaxis assay.

### Methylotaxis MCP homologs in *Methylobacterium* genomes

We sought MCP genes in the genomes of 62 *Methylobacterium* type strains using 52 amino acid sequences of strain 22A MCPs by BlastP (40% identity threshold). We found 1,467 MCP genes in total. MCPs with more than 60% identity to strain 22A methylotaxis MCPs were counted as methylotaxis MCP homologs.

### Statistical analysis

The statistical analysis of data (Student’s *t-*test and analysis of variance) was carried out using Prism 6 (GraphPad Software, Inc., CA, USA).

## Data availability statement

The original contributions presented in the study are included in the article/Supplementary material, further inquiries can be directed to the corresponding author.

## Author contributions

AT: Conceptualization, Data curation, Formal analysis, Funding acquisition, Investigation, Methodology, Project administration, Resources, Supervision, Validation, Visualization, Writing – original draft, Writing – review & editing. SM: Conceptualization, Investigation, Methodology, Writing – review & editing. YF: Investigation, Methodology, Writing – review & editing. TI: Investigation, Methodology, Writing – original draft, Writing – review & editing. YH: Investigation, Methodology, Writing – original draft, Writing – review & editing. SKi: Investigation, Methodology, Writing – original draft, Writing – review & editing. WS: Investigation, Methodology, Writing – original draft, Writing – review & editing. HL: Investigation, Methodology, Writing – original draft, Writing – review & editing. SKa: Investigation, Methodology, Writing – original draft, Writing – review & editing. HY: Conceptualization, Investigation, Methodology, Writing – original draft, Writing – review & editing. YS: Conceptualization, Investigation, Supervision, Writing – original draft, Writing – review & editing. JK: Conceptualization, Investigation, Methodology, Writing – original draft, Writing – review & editing.
