# Supplementary material for: Metabolism-linked methylotaxis sensors responsible for plant colonization in Methylobacterium aquaticum strain 22A
Source: Front Microbiol. 2023 Oct 13;14:1258452. doi: 10.3389/fmicb.2023.1258452 (PMC10613068; doi:10.3389/fmicb.2023.1258452)
Supplement: Supplementary file 3 [file Data_Sheet_1.pdf]

Supplementary figures Tani et al.

Metabolism-linked methylotaxis sensors responsible for plant colonization in *Methylobacterium aquaticum* strain 22A

Fig. S1.

A. Methylotaxis of strain 22A wild type grown in different conditions. The strain 22A wild type was grown on solid MM containing 0.5% methanol in the presence (open circle) /absence (filled circle) of 1 μM $LaCl_3$, 0.5% succinate (square), or R2A (triangle). The data are presented as the mean ± standard deviation (SD) (n = 3).

B. Effect of incubation time prior to chemotaxis assay. Strain 22A grown on 0.5% methanol in the absence of $LaCl_3$ for 1 day was suspended and incubated in HEPES buffer at 20°C for a varied time prior to methylotaxis assay. The data are shown as the mean rate ± SD (n = 3).

C. Effect of incubation temperature prior to chemotaxis assay. Strain 22A grown on 0.5% methanol in the absence of $LaCl_3$ was suspended and incubated in HEPES buffer at different temperatures for 2 h prior to methylotaxis assay. The data are presented as the mean ± SD (n = 3).

D. Chemotaxis for methanol, DL-malate, DL-glycerate, and L-glutamine of strain 22A, grown on methanol in the absence of $LaCl_3$. The data are presented as the mean ± SD (n = 3).

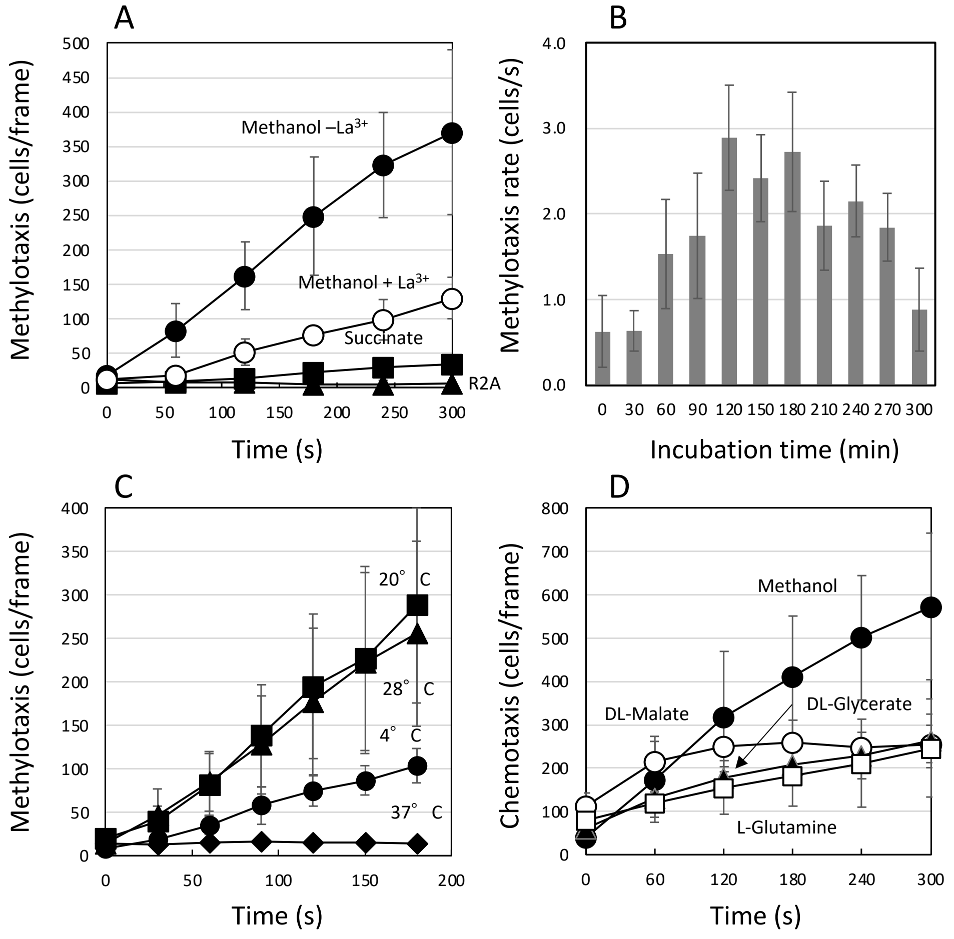

Metabolism-linked methylotaxis sensors responsible for plant colonization in *Methylobacterium aquaticum* strain 22A

Fig. S2.

Schematic presentation of the motifs in 52 MCPs encoded in strain 22A genome. Motifs were scanned by NCBI (https://www.genome.jp/tools/motif/), using the Pfam database with E-value 0.001. The MCPs indicated by asterisks were knocked out in this study. Red, dCache (double CAlcium channels and CHEmotaxis receptors) domain; yellow, HAMP (Histidine kinases, Adenylate cyclases, Methyl-accepting proteins, and Phosphatases) domain; black, PF00015 Methyl-accepting chemotaxis protein (MCP) signaling domain; blue, 4HB_MCP_1 (Four Helix Bundle sensory module for signal transduction); CHASE3, CHASE3 (Cyclases/Histidine kinases Associated Sensory Extracellular) domain; pilZ, PilZ (bacterial type IV pilus assembly) domain; PAS and PAS_3, PAS (Per ARNT Sim) fold; Protoglobin, protoglobin; Snoal_2, Snoal-like polyketide cyclase domain.

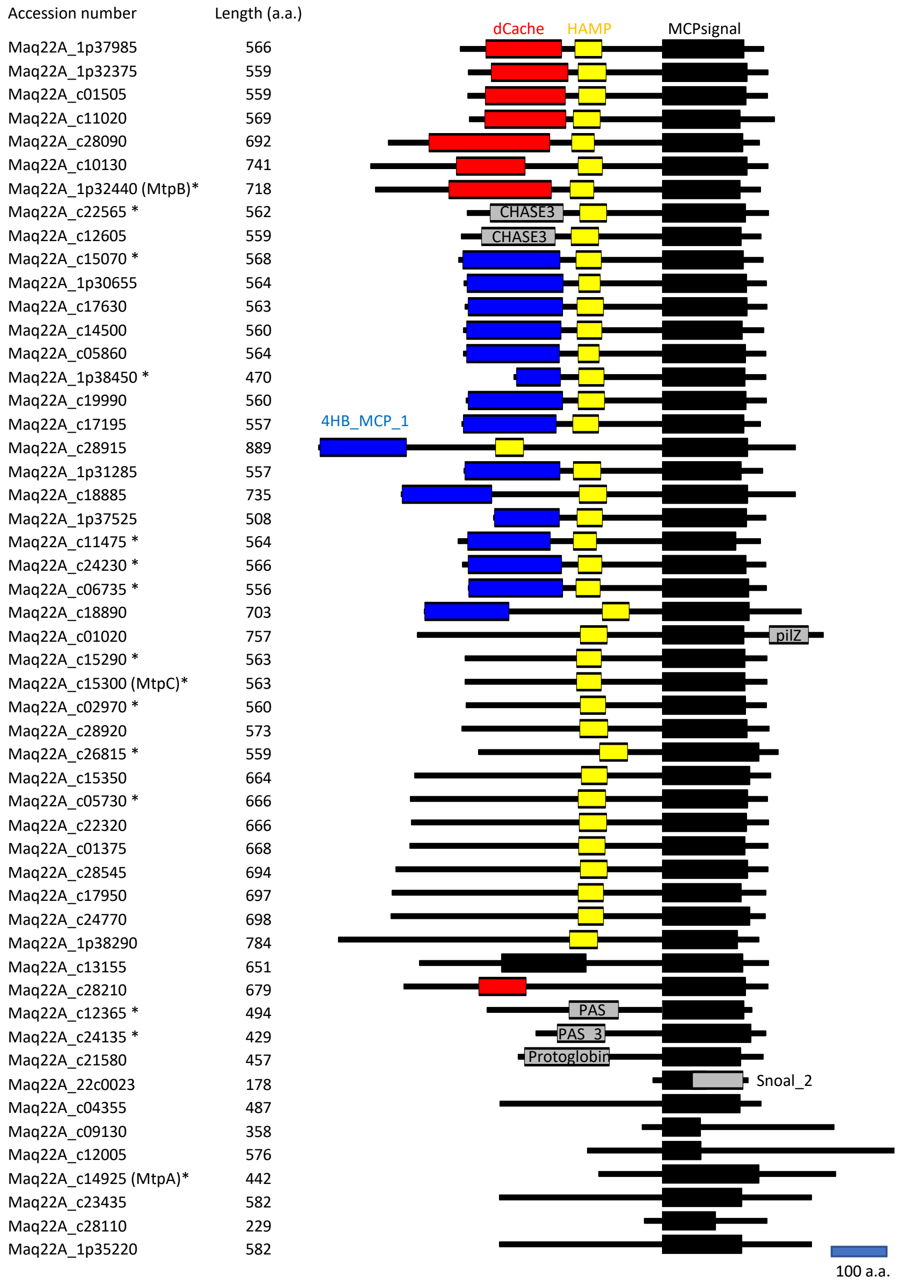

Metabolism-linked methylotaxis sensors responsible for plant colonization in *Methylobacterium aquaticum* strain 22A

Fig. S3.

Left, expression level of 52 MCP genes in strain 22A. The MCPs indicated by asterisks were knocked out in this study. The data are from the RNA-Seq data reported previously (Masuda et al., 2018). Expression level is shown as log 2 scale of FPKM values. Strain 22A wild type was grown on succinate, or methanol in the presence of 30 μM $CaCl_2$, 30 μM $LaCl_3$, and 30 μM $CaCl_2$ plus 30 μM $LaCl_3$. The data are visualized with heatmap.2 package in R (R core Team, 2018). Right, methylotaxis rate of single MCP gene mutants measured in the screening process. The data were analyzed with the Student's *t*-test against wild type data and are shown as mean ± standard deviation (n = 3~6).

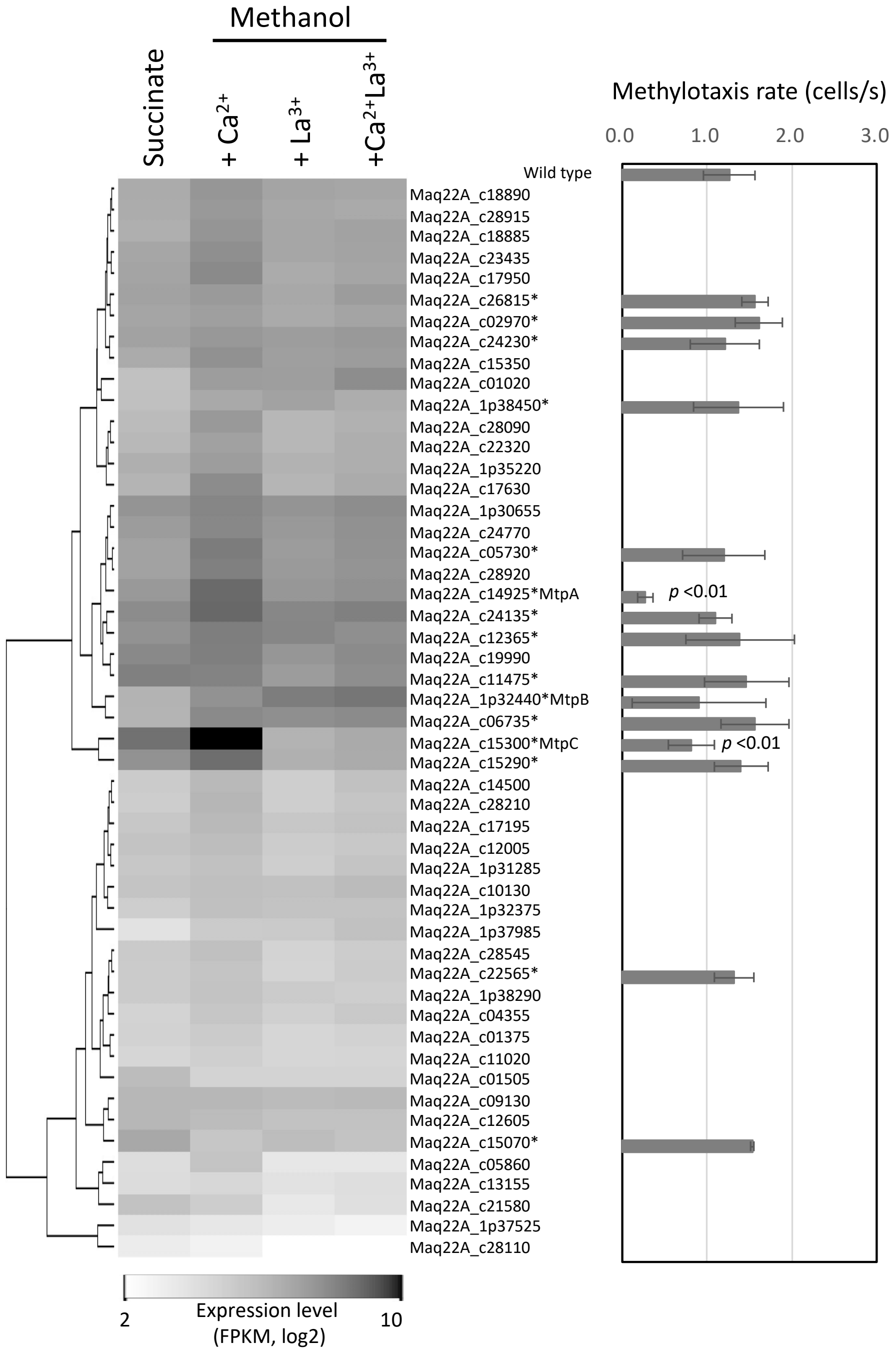

Fig. S4.

Methylotaxis in strain 22A TM carrying pAT01-Mtp1, -Mtp2, and -Mtp3 grown on methanol in the presence/absence of $LaCl_3$. The data were analyzed with the Student's *t*-test and are shown as the mean rate ± standard deviation (n = 3).

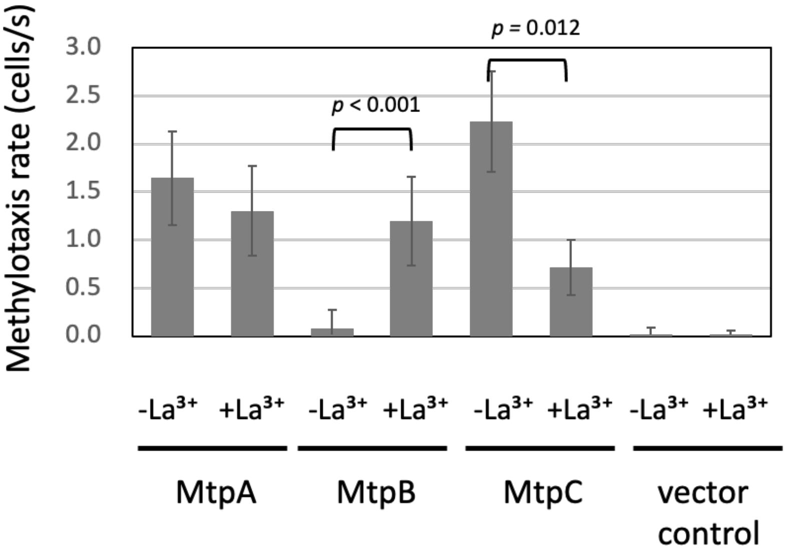

Fig. S5.

Growth of strain 22A wild type and TM on methanol and succinate. The cells were grown in 200 µl media prepared in 96-well plates at 28°C. (A) Growth on 0.5% methanol. (B) Growth on 0.5% succinate. Circles, wild type; rectangles, TM; closed symbols, in the absence of La^{3+}; open symbols, in the presence of La^{3+}. The data are shown as the mean ± standard deviation (n = 3).

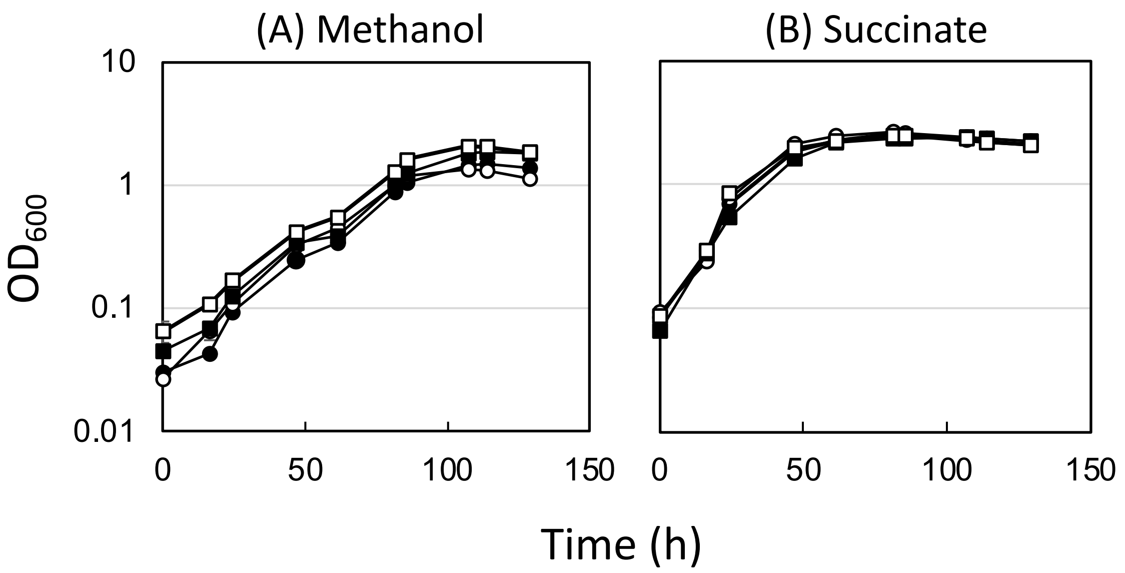

Fig. S6.

A. Experimental setup. The root of a sterile rice seedling (7 days old) was put in 18 ml Kimura B nutrient solution (Ma et al., 2001) in a 50 ml tube containing 35 ml glass beads (diameter, 6 mm), and allowed to grow at 26°C with a light/dark cycle of 14/10 h, respectively, 135 μmol·m^{-2}·s^{-1}. One microliter of the culture medium was analyzed with a gas chromatograph (GC-2014, Shimadzu) equipped with an InterCap WAX column (0.53 mm × 30 m; GL Sciences Inc.) and a flame ionization detector, under the following conditions: injection temperature, 230°C; detector temperature, 250°C; column temperature, 110–152°C (6°C/min for 7 min and 152°C for 3 min); and flow rate of the carrier gas (N_2), 10 ml/min. A control experiment was carried out without rice seedlings.

B. Quantification of methanol released from rice root in the hydroponic culture. The size of the plants used in the experiment was 0.07 ± 0.01 g (fresh weight), 11.28 ± 1.05 cm (shoot length), and 8.58 ± 1.71 cm (root length), on average, at the end of the experiment (5 d). The data are shown as the mean ± standard deviation (n = 3).

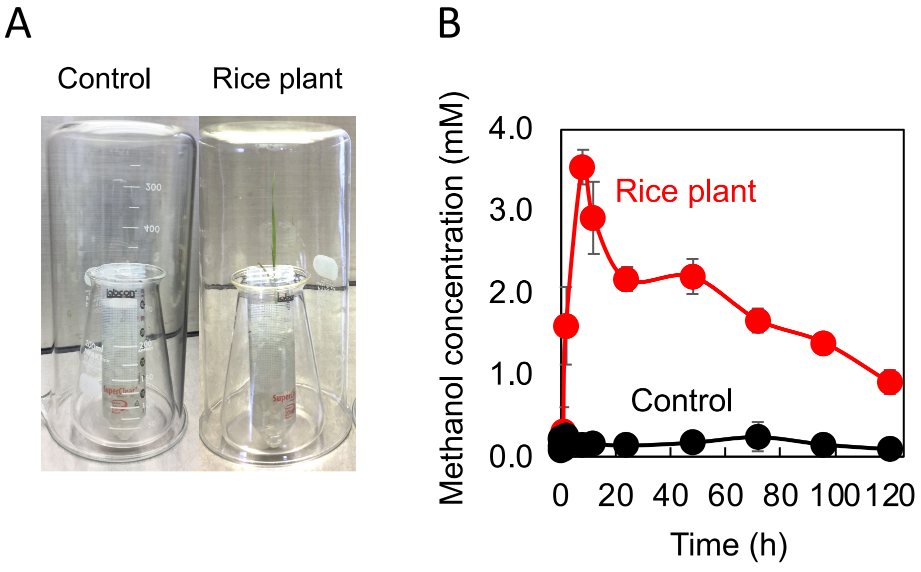

Fig. S7.

Formtaxis in strain 22A TM carrying pCM130KmC-Mcp1-GFP (shown as TM+MtpA-GFP), grown on methanol in the presence/absence of $LaCl_3$. The data were analyzed with the Student's *t*-test and are shown as the mean rate ± standard deviation (n = 3).

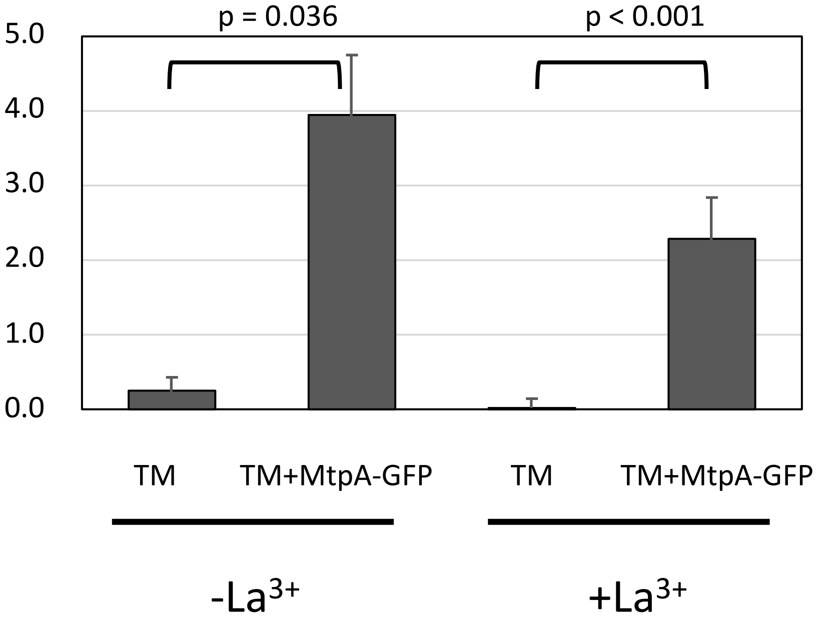

Fig. S8.

A. Chemotaxis toward 2% ethanol of the strain 22A wild type and MCP knockout mutants grown on methanol in the presence/absence of $LaCl_3$. The data are shown as the mean rate ± standard deviation (SD) (n = 3).

B. Methylotaxis in strain 22A grown on methanol in the presence/absence of $LaCl_3$ in the presence of varied concentrations of ethanol. The data are shown as the mean rate ± SD (n = 3).

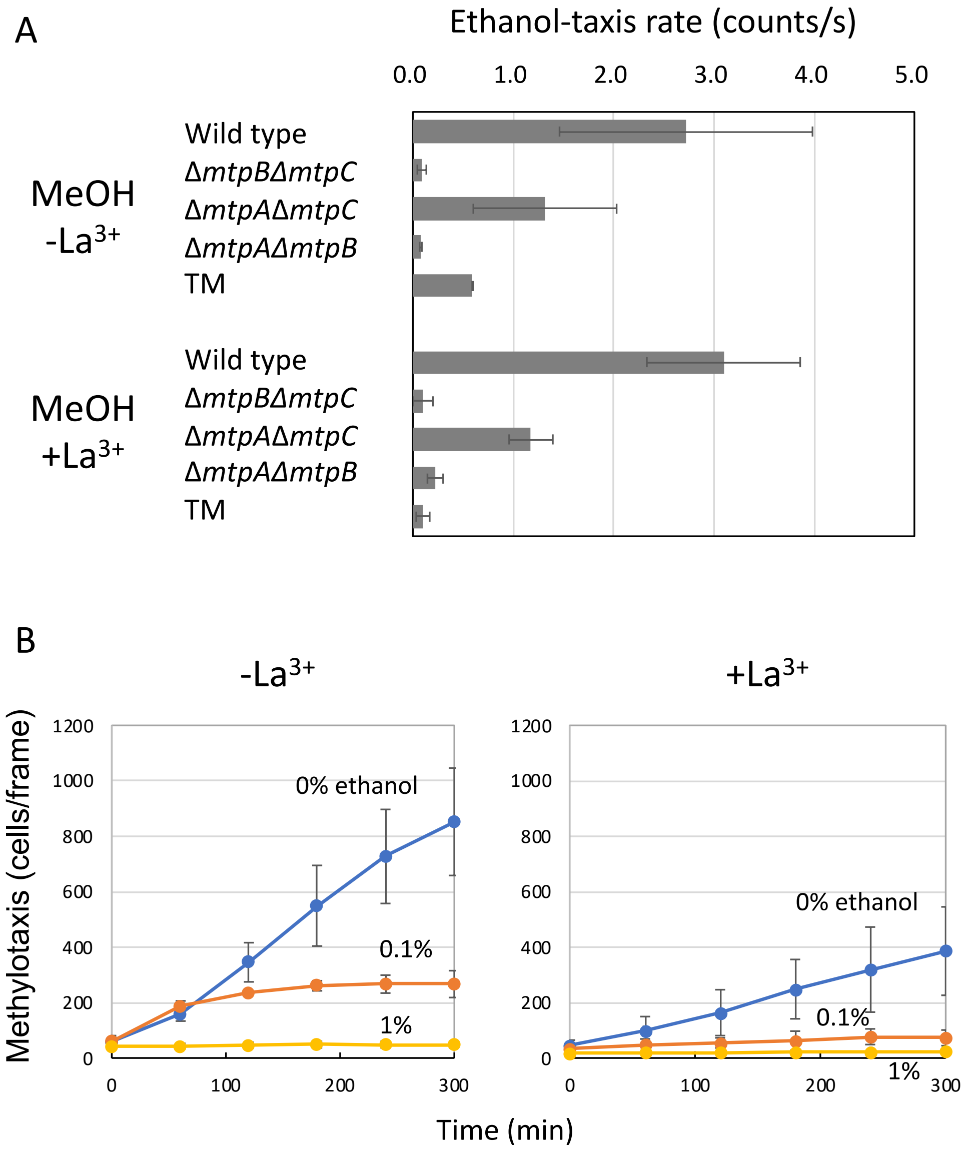

References

Ma JF., Goto S., Tamai K., Ichii M. 2001. Role of root hairs and lateral roots in silicon uptake by rice. Plant Physiol 127, 1773-1780.

Masuda S, Suzuki Y, Fujitani Y, Mitsui R, Nakagawa T, Shintani M, Tani A. 2018. Lanthanide-dependent regulation of methylotrophy in *Methylobacterium aquaticum* strain 22A. mSphere. 3(1):e00462-17. doi: 10.1128/mSphere.00462-17.

R Core Team 2018. R: A language and environment for statistical computing. R Foundation for Statistical Computing, Vienna, Austria. URL https://www.R-project.org/.
